# Supplementary material for: Comparative Transcriptome Analysis between a Resistant and a Susceptible Wild Tomato Accession in Response to Phytophthora parasitica
Source: Int J Mol Sci. 2018 Nov 23;19(12):3735. doi: 10.3390/ijms19123735 (PMC6320849; doi:10.3390/ijms19123735)
Supplement: Supplementary file 1 [file ijms-19-03735-s001.zip › ijms-391324-supplementary-proofdone/Afzaletal_Figures_Tomato_ReferenceMapping_figures.pdf]

e-Xtra

Comparative transcriptome analysis between a resistant and a susceptible wild tomato accession in response to *Phytophthora parasitica*

Zunaira Afzal Naveed [1,2] and Gul Shad Ali[1,2]*

[1]Mid-Florida Research and Education Center and [2]Department of Plant Pathology, University of Florida/Institute of Food and Agricultural Sciences, Apopka, FL, USA.

Corresponding Author:

Gul Shad Ali*

gsali@ufl.edu

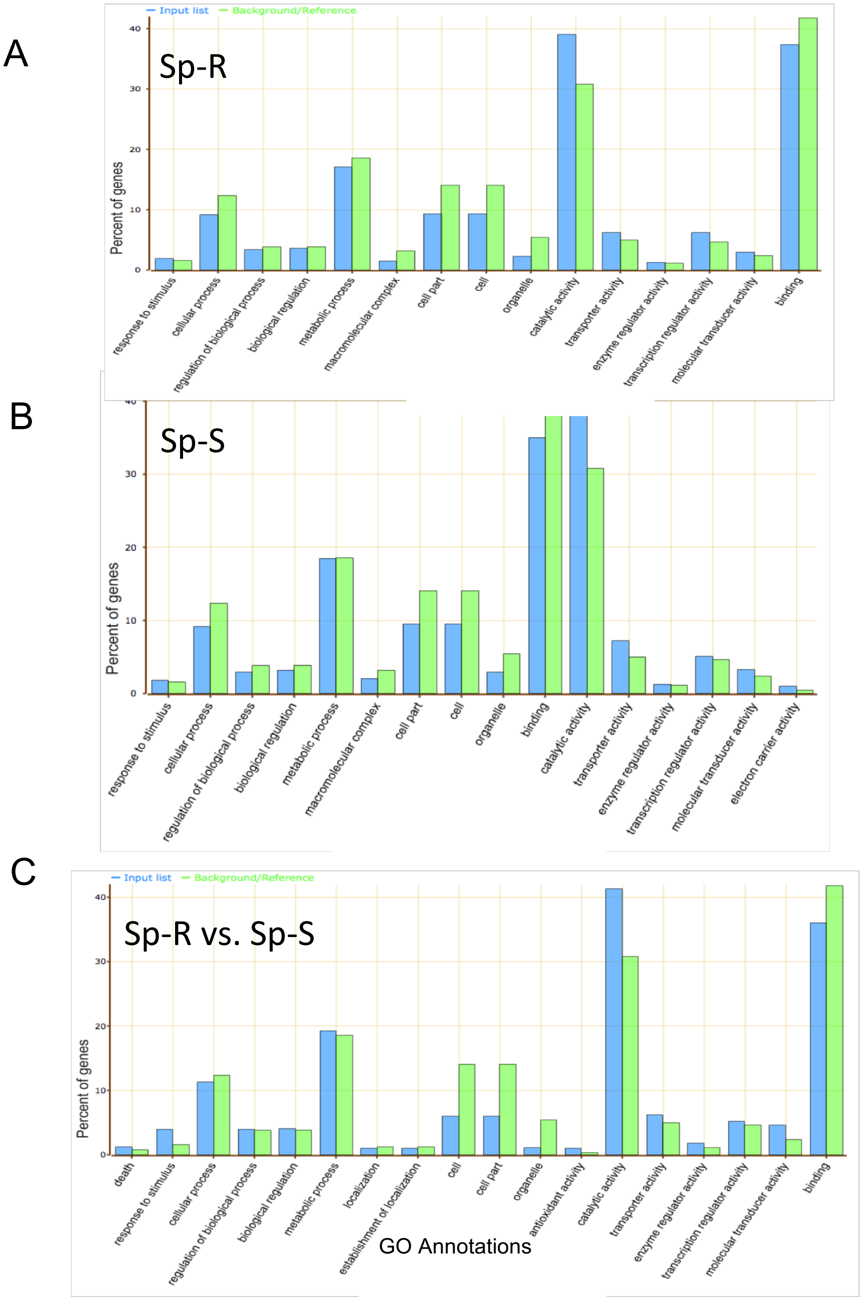

A
Input list
Background/Reference
Sp-R
Percent of genes
0
10
20
30
40
response to stimulus
cellular process
regulation of biological process
biological regulation
metabolic process
macromolecular complex
cell part
cell
organelle
catalytic activity
transporter activity
enzyme regulator activity
transcription regulator activity
molecular transducer activity
binding
B
Sp-S
Percent of genes
0
10
20
30
40
response to stimulus
cellular process
regulation of biological process
biological regulation
metabolic process
macromolecular complex
cell part
cell
organelle
binding
catalytic activity
transporter activity
enzyme regulator activity
transcription regulator activity
molecular transducer activity
electron carrier activity
C
Input list
Background/Reference
Sp-R vs. Sp-S
Percent of genes
0
10
20
30
40
death
response to stimulus
cellular process
regulation of biological process
biological regulation
metabolic process
localization
establishment of localization
cell
cell part
organelle
antioxidant activity
catalytic activity
transporter activity
enzyme regulator activity
transcription regulator activity
molecular transducer activity
binding
GO Annotations

Supplementary Figure 1. Overview of GO annotations assignment of DEGs by SEA tool of AgriGO. Percent distribution of DEGs across enriched GO terms for cellular component, molecular function and biological processes for all DEGs found in *P. parasitica* treated vs. control samples of Sp-R genotype at both 24 and 48hpi (A) for Sp-S genotype (B) DEGs identified by Sp-R treated vs. Sp-S treated samples at both time points (C). Green bars represents reference background (*S. lycopersicum* ITAG 2.4) used to calculate enrichment and blue bar represent input DEGs.

Sp-R

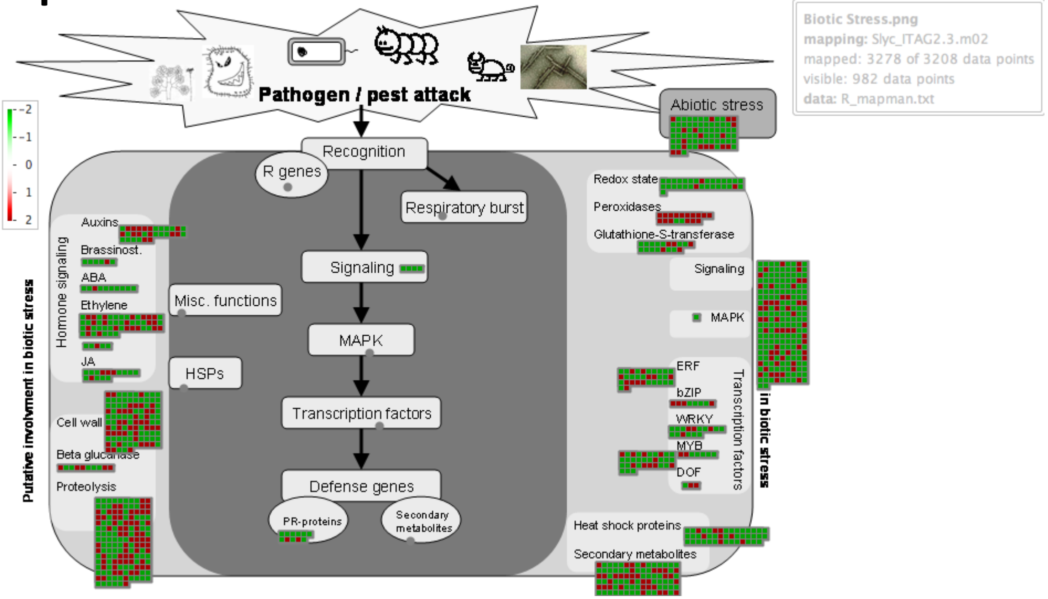
Pathogen / pest attack
Abiotic stress
Biotic Stress.png
mapping: Slyc_ITAG2.3.m02
mapped: 3278 of 3208 data points
visible: 982 data points
data: R_mapman.txt
-2
-1
0
1
2
Putative involvment in biotic stress
Hormone signaling
Auxins
Brassinost.
ABA
Ethylene
JA
Cell wall
Beta glucanase
Proteolysis
Recognition
R genes
Respiratory burst
Signaling
Misc. functions
MAPK
HSPs
Transcription factors
Defense genes
PR-proteins
Secondary metabolites
Redox state
Peroxidases
Glutathione-S-transferase
Signaling
MAPK
ERF
bZIP
WRKY
MYB
DOF
Transcription factors
in biotic stress
Heat shock proteins
Secondary metabolites

Sp-S

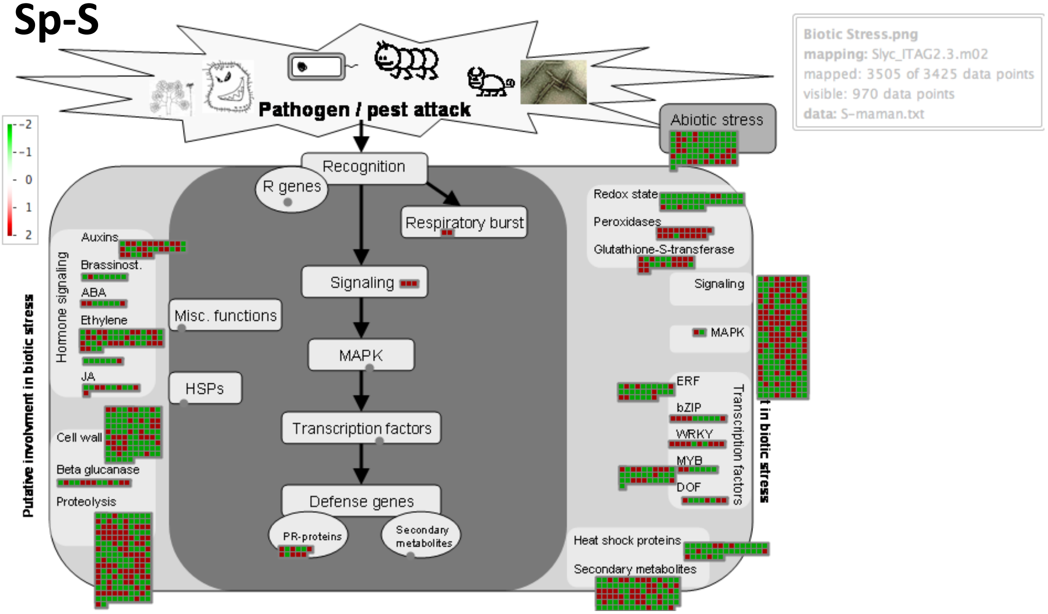
Pathogen / pest attack
Abiotic stress
Biotic Stress.png
mapping: Slyc_ITAG2.3.m02
mapped: 3505 of 3425 data points
visible: 970 data points
data: S-maman.txt
-2
-1
0
1
2
Putative involvment in biotic stress
Hormone signaling
Auxins
Brassinost.
ABA
Ethylene
JA
Cell wall
Beta glucanase
Proteolysis
Recognition
R genes
Respiratory burst
Signaling
Misc. functions
MAPK
HSPs
Transcription factors
Defense genes
PR-proteins
Secondary metabolites
Redox state
Peroxidases
Glutathione-S-transferase
Signaling
MAPK
ERF
bZIP
WRKY
MYB
DOF
Transcription factors
t in biotic stress
Heat shock proteins
Secondary metabolites

Supplementary Figure 2: Visualization of differentially expressed genes in wild tomatoes in response to *P. parasitica* on biotic stress pathway , (A) Resistant accession (B) Susceptible accession. Color gradient represents log2 fold ratios with red representing upregulation and blue representing downregulation in treatments over controls.

Sp-R

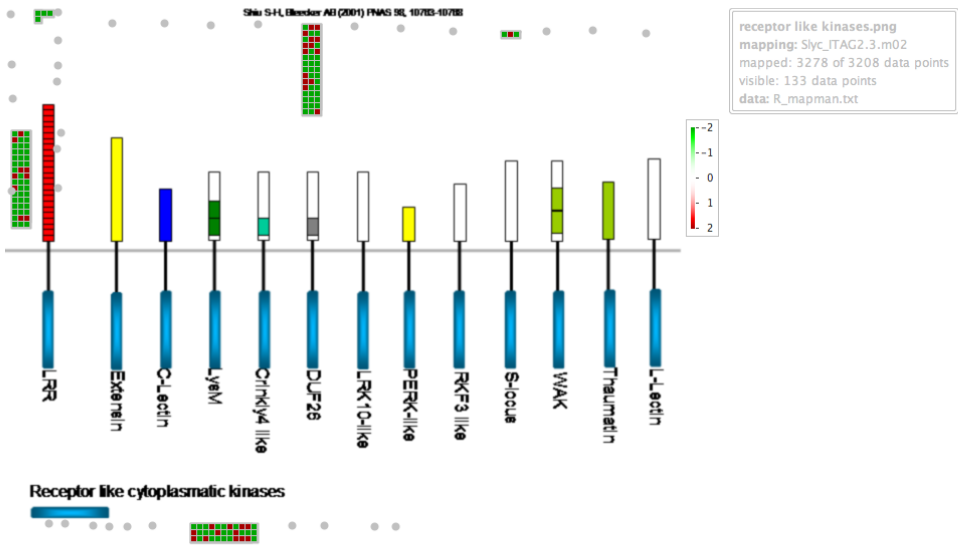
Shiu S-H, Bleecker AB (2001) PNAS 98, 10763-10768
receptor like kinases.png
mapping: Slyc_ITAG2.3.m02
mapped: 3278 of 3208 data points
visible: 133 data points
data: R_mapman.txt
-2
-1
0
1
2
LRR
Extensin
C-Lectin
LysM
Crinkly4 like
DUF26
LRK10-like
PERK-like
RKF3 like
S-locus
WAK
Thaumatin
L-Lectin
Receptor like cytoplasmatic kinases

Sp-S

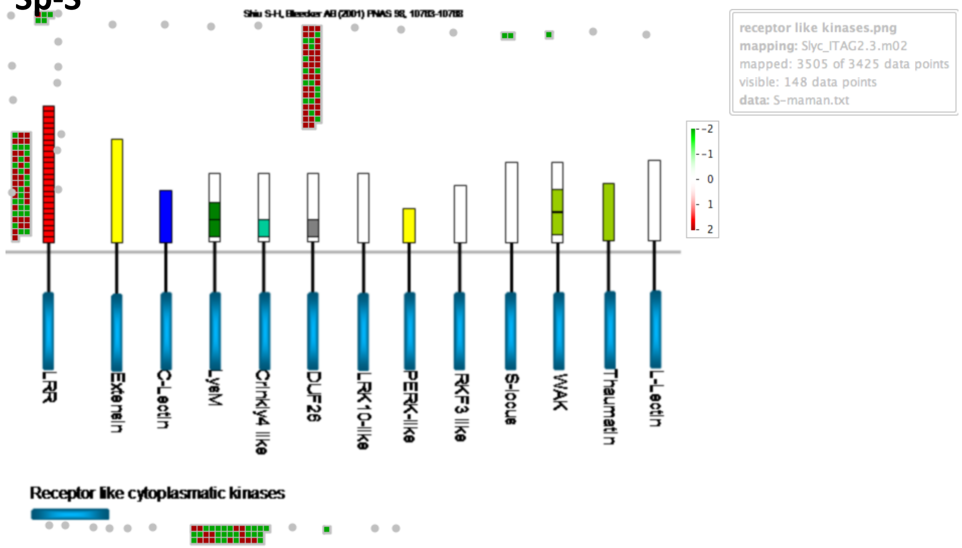
Shiu S-H, Bleecker AB (2001) PNAS 98, 10763-10768
receptor like kinases.png
mapping: Slyc_ITAG2.3.m02
mapped: 3505 of 3425 data points
visible: 148 data points
data: S-maman.txt
-2
-1
0
1
2
LRR
Extensin
C-Lectin
LysM
Crinkly4 like
DUF26
LRK10-like
PERK-like
RKF3 like
S-locus
WAK
Thaumatin
L-Lectin
Receptor like cytoplasmatic kinases

Supplementary Figure 3 : Differentially expressed genes of wild tomatoes in response to *P. parasitica*, encoding DUF26 type receptor like kinases (A) Resistant accession (B) Susceptible accession. Color gradient represents log2 fold ratios with red representing upregulation and blue representing downregulation in treatments over controls.

Table S1. qRT-PCR primers for selected genes.

Transcript ID	Description	Primers
**Solyc07g043420.2**	2-oxoglutarate and Fe dependent oxygenase	GGAGGCAGGGTTCTTTGACA
		ATGCATTGCGAATAGGCTGC
**Solyc08g029000.2**	lipoxygenase 1	AGCACACCCGATGAGTTTGA
		TGAAGAACTTGAGGTGTTGGGA
**Solyc09g010630.2**	heat shock protein 70	ATGGCTGGAAAGGGTGAAGG
		ACGCTCAGAGTCGGTGAATC
**Solyc11g069700.1**	Elongation factor Tu family	TGTGCCGATTTCTGGTTTCG
		ACGACCAACAGGAACAGTCC
**Solyc04g080960.2**	Papain family cysteine protease	GACTGGCGTGAAAAAGGAGC
		GGGTCACACTCATGGTCACA

Table S2: Number of DEGs found among all comparisons.

	Total DEGs	Up regulated DEGs	Down regulated DEGs	Specific DEGs
S-24hpi treated vs S-control	3079	1132	1947	775
S-48hpi treated vs S-control	1919	811	1108	152
R-24hpi treated vs R-control	2657	868	1789	519
R-48hpi treated vs R-control	1836	606	1230	293
	4598*			
R vs. S				
R-control vs S-control	322	155	167	178
R-24hpi treated vs S-24hpi treated	1158	287	871	589
R-48hpi treated vs S-48hpi treated	889	207	682	302
	1681*			
